# The birth, life, and death of a cytopathology board examination question

**DOI:** 10.1002/cncy.70049

**Published:** 2025-09-27

**Authors:** Roseann I. Wu, Ritu Nayar, Gary W. Procop

**Affiliations:** ^1^ Department of Pathology and Laboratory Medicine Penn Medicine Philadelphia Pennsylvania USA; ^2^ Department of Pathology Northwestern Medicine Chicago Illinois USA; ^3^ American Board of Pathology Tampa Florida USA

**Keywords:** American Board of Pathology (ABPath), cytopathology, examination, National Board of Medical Examiners (NBME), testing

Have you ever wondered how a particular cytopathology question ended up on your anatomic pathology primary certification examination or the cytopathology subspecialty certification examination? This is how it happens.

The validity of high‐stakes certifications, such as those administered by the American Board of Pathology (ABPath), is supported by psychometric evidence.^1^ The qualifications of the individuals who write and review the examination items (i.e., questions) and the process by which the items are reviewed, edited, improved upon, curated, and eventually eliminated are of paramount importance to the validity of the examination.^2^

## Selection of item‐writing committee members

The item‐writing committees of the ABPath are referred to as Test Development and Advisory Committees (TDACs) because they both create, review, edit, and eliminate items as well as provide the ABPath with guidance on topics within their scope of expertise. Examples of instrumental guidance that the TDACs have provided include the population and maintenance of the Guidelines and Key Manuscripts and the ABPath Content Specifications resources. These groups also review and update the ABPath Examination Blueprints each year during their committee meetings.

Item writers consist predominantly of nationally recognized subspecialty experts who are engaged in teaching at their respective institutions and are academically productive. TDAC members are selected from a list of individuals who meet these criteria, and members of the pathology community who meet these criteria may request inclusion via the ABPath website. Cytopathology TDAC members must be board‐certified pathologists and are preferably but not necessarily boarded in cytopathology. Although TDAC members are not permitted to give board review lectures or create test preparation materials for 6 years after their term ends, individuals with prior involvement in these activities are not excluded from TDAC membership.

Committee members are expected to attend in‐person meetings, submit the requisite number of items for consideration, meet deadlines for item submission, and actively participate during meetings. Active participation includes engaging in a collegial but robust discussion regarding the item content, item anatomy (i.e., how the item is written), item relevance, item difficulty level, and technical item‐related issues (e.g., assignment of the appropriate category code). The selection of TDAC members is based on the needs of the committee and the characteristics of the individual. Members of the TDAC, in addition to other selected volunteers, also participate in standard‐setting activities every 4 years or so. TDAC members are reappointed on an annual basis with consideration given to previous performance and may serve for a maximum of 6 years.

## Item creation

The ABPath staff performs a search of the ABPath Item Bank in the relevant area before each TDAC meeting. This report is provided to the TDAC chair, who denotes the areas within the Item Bank that could benefit from additional items. This information, as well as the experience from the previously assembled examinations, is used to identify areas where new items are needed. The TDAC chair, in conjunction with ABPath staff, will then request the needed items with consideration to the members of the TDAC and their respective areas of subspecialty expertise.

TDAC members are expected to create items while following instructions provided by the ABPath, which follow the National Board of Medical Examiners single best‐answer format. The items consist of written (recall) items, practical interpretation items, and items that use virtual microscopy. Written items that necessitate the recall of facts are minimized, with an emphasis on the other two item types; factual recall assesses “knowledge,” which is the base of the Miller pyramid (i.e., “knows”).^3^ Practical items require more advanced knowledge, application of that knowledge, and interpretation, which is the next level (i.e., “knows how”) of the Miller pyramid. Complex practical items and virtual microscopy items assess “shows how,” the third level of the Miller pyramid, which begins competency assessment. Practical items include image, graph, and text interpretation, and may include second‐order items. For example, a first‐order question is usually a recognition question (e.g., recognition of a “decoy” cell), whereas a second‐order question would ask for the cause of the depicted cytomorphologic changes. The test taker would need to not only recognize the decoy cell but also know that the cytomorphologic changes were caused by polyoma virus infection. The virtual microscopy slides are digitized glass slides viewed on a computer screen. The candidate is required to review the virtual slide, interpret the contents of the slide, which requires navigating to areas of the slide that optimally show diagnostic material (i.e., locator skills), adjust the magnification, and then select the correct response (i.e., diagnostic skills). The Virtual Microscopy section consists of both knowledge and competency assessments.

The item writer is initially responsible for the accuracy of the item content. The use of artificial intelligence/large language models (AI/LLMs) is prohibited in the construction of items for the primary and subspecialty certification examinations. Because of the high‐stakes nature of these examinations, it is important for the validity of these examinations to demonstrate content generation from a subject matter expert and undergo subsequent vetting by a group of content experts. Additionally, ABPath copyright protects the contents of the primary and subspecialty examinations, and currently, AI/LLM‐generated items cannot be protected by US copyrights. The newly formed items need to be aligned with ABPath Content Specifications.

## Item review, editing, approval, or elimination

Items are entered by the TDAC member into a secure, ABPath‐constructed electronic notebook. The electronic notebook allows TDAC members to securely enter their proposed items, as well as review items from other TDAC members and offer recommendations for improvement. When the TDAC members meet in person, the ABPath TDAC coordinator presents each item one at a time on a mutually viewed screen, and all TDAC members suggest/discuss edits and debate the necessity of the item on a certification examination. Most items are collectively edited in some manner, whereas a minority will be rejected as unsuitable for the proposed examination. Photomicrographs and the selection of suitable virtual slides can be particularly challenging in cytopathology, given the three dimensionality of cytologic slide preparations and variable smear thickness. The item’s difficulty level, category code, feedback diagnosis, and anatomic site are verified, and a reference that supports the item is documented. Items that make it through this gauntlet have made it into the ABPath Item Bank for potential use in a certification examination.

## Ongoing item review, retention, or elimination

If an item is selected for inclusion on an examination, that item will generate statistics solely on the basis of its performance with respect to the candidates taking the examination. Items with acceptable performance statistics are kept in the Item Bank for potential use in the future. Items with unacceptable statistical parameters are marked for Test Committee Review (TCR). During examination construction by the TDAC chair, items may also be marked for TCR if there is any possibility that the item is no longer valid because of, for instance, updated guidelines or nomenclature (e.g., “Atypical Glandular Cells of Undetermined Significance” or “Hürthle cell neoplasm”). Candidates for certification can provide anonymous feedback on items; items that are flagged by at least four candidates are sent for TCR. Items with poor images are also flagged for review; the ABPath has made a concerted effort to improve the quality of images on the examinations.

**FIGURE 1:**
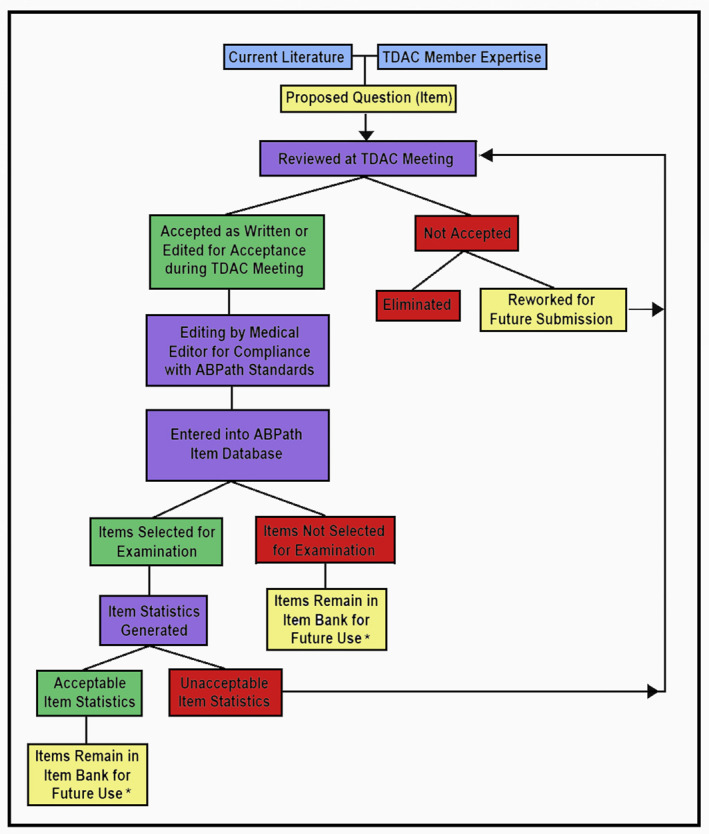
“Life cycle” of an ABPath test item. *The Test Committee Review process and periodic Item Bank review are used to purge the Item Bank of outdated and/or problematic items. ABPath indicates American Board of Pathology; TDAC, Test Development and Advisory Committee.

During part of their meeting, TDAC members will review all “TCR items,” as well as the associated statistics, and will either update and/or fix the item or eliminate it from the Item Bank. Finally, sections of the Item Bank are curated on a periodic basis to remove items that are outdated and review items that have been entered into the Item Bank but were never used. This periodic curation, in conjunction with a robust TCR process and new item incorporation process, helps keep the Item Bank of the ABPath current and relevant (Figure 1).

## Future directions

The ABPath has made significant efforts to promote transparency and provide guidance to trainees with the publication of the ABPath Content Specifications and Guidelines and Key Manuscripts. The ABPath Histology Primer was designed and created to be a free educational resource for those entering our field as well as to assist pathology program directors. The ABPath is engaged with our stakeholders in supporting the optimal training and development of future generations of pathologists. As a result, the ABPath has formalized its support of competency‐based medical education and adopted a resolution on a competency‐based assessment (CBA) strategy (ABPath Resolution on CBA Strategy). The ABPath is also exploring alternate forms of assessment, including simulation that more closely approximates what a pathologist does in practice. Newer assessment strategies that evaluate judgment in clinical scenarios and the appropriate utilization of medical resources are also under consideration. The ABPath, as a member of the American Board of Medical Specialties, is collaborating with other specialty boards and the ABPath’s Cooperating Societies on these endeavors. Individuals who are interested in contributing to item generation for the ABPath are encouraged to volunteer as item writers for the ABPath CertLink Program.

## Referenced websites

ABPath Guidelines and Key Manuscripts: https://abpath.org/guidelines‐and‐key‐manuscripts/

ABPath Content Specifications: https://abpath.org/content‐specifications‐for‐examinations/

ABPath Histology Primer: https://histologyprimer.abpath.org/

ABPath Examination Blueprints: https://abpath.org/exam‐blueprints/ and https://abpath.org/exam‐blueprints‐and‐daily‐exam‐schedules‐ss/

ABPath Resolution on Competency‐Based Assessment Strategy: https://abpath.org/resolution‐competency‐based‐assessment/

ABPath’s Cooperating Societies: https://abpath.org/cooperating‐societies/

ABPath CertLink Program: https://abpath.org/contribute‐questions/
